# Metagenomic insights into postbiotic-mediated modulation of strawberry surface microbiome and metabolic activity

**DOI:** 10.3389/fmicb.2026.1841388

**Published:** 2026-05-28

**Authors:** Gabriela N. Tenea, Pablo Jarrín-V., Pamela Reyes

**Affiliations:** 1 Biofood and Nutraceutics Research and Development Group, Faculty of Engineering in Agricultural and Environmental Sciences, Universidad Tecnica del Norte, Ibarra, Ecuador; 2 Dirección de Innovación, Instituto Nacional de Biodiversidad, Quito, Pichincha, Ecuador

**Keywords:** antimicrobials, microbiome profile, pathway profile, postbiotics, shotgun metagenomics, strawberries

## Abstract

**Introduction:**

The increasing demand for sustainable alternatives to chemical disinfectants in postharvest fruit handling has incentivized exploration into microbiome-based interventions. We evaluated the impact of lactic acid bacteria (LAB)-derived postbiotic formulations (FF1, FF2, FF3) and a commercial disinfectant (CD) on the microbial community structure of the strawberry fruit surface.

**Methods:**

Taxonomic and functional changes in the microbial communities were evaluated using shotgun metagenomic sequencing, enabling comprehensive profiling of microbial composition and functional potential through gene family abundance, EggNOG functional categories, KEGG pathways, and MetaCyc metabolic reconstruction. The tested formulations consisted of a precipitated peptide-protein extract (PP) from *Weissella cibaria* UTNGt21O (FF2), used as the antimicrobial agent, and an exopolysaccharide (EPS) from *W. confusa* UTNCys2-2 (FF3), serving as a biopolymer carrier, applied in combination (FF1: PPGt21O + EPSCys2-2) or individually.

**Results:**

Our integrated analysis revealed that the highly suppressive formulation, FF1, outperformed the CD by fundamentally restructuring the microbial landscape. Taxonomically, FF1 notably reduced the abundance of key opportunistic spoilage or hazardous organisms. Rather than acting as an indiscriminate biocide, FF1 functioned as a targeted ecological disruptor. Functional profiling (eggNOG, KEGG, and MetaCyc) suggested potential shifts in functional capacity, including a reduced relative abundance of genes associated with translation machinery, cellular membrane expansion (stearate biosynthesis), and host lipid degradation (fatty acid β-oxidation). In parallel, the FF1-treated microbiome showed a higher relative abundance of genes linked to stress-response functions, including heat shock proteins and cell wall-related processes such as peptidoglycan maturation. In contrast, less restrictive formulations (FF2 and FF3) permitted the proliferation of opportunists such as *Pseudomonas* spp. and *Xanthomonas fragariae*, accompanied by active energy-consuming and tissue-degrading metabolic signatures.

**Conclusion:**

These findings suggest possible underlying mechanisms of LAB-derived postbiotics, demonstrating that FF1 forces the surface microbiome into a metabolically restricted, non-degradative survival state, potentially contributing to the preservation of postharvest strawberry quality.

## 1 Introduction

Fresh fruits, particularly strawberries, are highly susceptible to microbial contamination throughout pre- and postharvest stages, owing to their elevated water activity, nutrient-rich tissues, and delicate epidermal structures (Priyadarshi et al., 2024a). These physicochemical characteristics create an ideal ecological niche for microbial colonization, proliferation, and spoilage (Founou et al., 2021; Rahman et al., 2021). Beyond quality degradation, microbial contamination poses serious food safety risks, especially when pathogenic or antibiotic-resistant bacteria (ARB) such as *Salmonella enterica*, *Escherichia coli*, and opportunistic yeasts like *Candida parapsilosis* are present on fruit surfaces (Tenea and Reyes, 2024; Tenea et al., 2024). This is of particular concern in market-sourced fruits, where handling, storage, and environmental exposure can exacerbate microbial presence and load (Gil et al., 2025; Simon et al., 2025). Conventional strategies to mitigate surface contamination commonly involve chemical sanitizers, which, despite their broad-spectrum efficacy in reducing microbial counts, often fail to eliminate resistant or stress-adapted species (Acevedo et al., 2024). In fact, these agents may inadvertently impose selective pressure, enriching for taxa harboring antimicrobial resistance genes (ARG) or mobile genetic elements (Allende and Monaghan, 2015; Olaimat and Holley, 2012). This limitation has catalyzed the search for alternative, microbiome-friendly interventions that can control pathogens without disrupting ecological balance (Haniffadli et al., 2024).

Among emerging approaches, postbiotics, non-viable microbial cells, metabolites, or cell components, offer a promising avenue for microbiome modulation and pathogen suppression without the biosafety concerns linked to live probiotics (Aguilar-Toalá et al., 2018). Unlike chemical sanitizers, postbiotics exert their effects through mechanisms such as membrane disruption, enzymatic degradation, and metabolic interference, potentially enabling selective suppression of undesirable taxa while promoting beneficial microorganisms (Mafe and Büsselberg, 2025; Tong et al., 2025). However, despite increasing recognition of their antimicrobial and health-promoting properties, the ecological consequences of postbiotic application on fruit-associated microbiota remain underexplored, particularly in the context of whole-community dynamics and functional capacity (Stelmach et al., 2024).

Over the past three years, food safety diagnostics and microbiome profiling have entered a disruptive revolution, transitioning from simple taxonomic cataloging to predictive, system-wide functional science. Metagenomic technologies, including shotgun and long-read sequencing, now provide high-resolution frameworks to characterize entire food microbiomes (Tenea et al., 2025a). As highlighted in recent food microbiology advances, these tools enable the mapping of functional genes to specific metabolic pathways (Tigrero-Vaca et al., 2025), the correlation of specific genera with functional KEGG pathways (Li et al., 2025), and the tracking of antimicrobial resistance genes (ARGs) across distinct ecological states (Zhao et al., 2025).

Recent metagenomic studies demonstrate that strawberry surfaces host dynamic microbial communities shaped by production and postharvest conditions (Olimi et al., 2022; Sangiorgio et al., 2022; Siegieda et al., 2024). Advances in high-throughput and shotgun metagenomics have improved taxonomic resolution and enabled functional profiling, revealing dominant field-associated taxa such as *Pseudomonas* spp. alongside postharvest opportunists including *Serratia liquefaciens* (Tenea and Reyes, 2024; Farkas et al., 2025). Recent work further highlights functional traits linked to stress tolerance, biofilm formation, and microbial interactions, as well as the contribution of fungal communities to fruit quality and resilience (Kwak et al., 2025; Son et al., 2026). Biopolymer-based edible coatings derived from polysaccharides, lipids, or proteins can limit microbial colonization and extend shelf life by acting as semi-permeable barriers that regulate gas exchange and water activity, thereby constraining microbial growth (Sogvar et al., 2016; Priyadarshi et al., 2024b). However, their broader application remains limited by challenges in scalability, cost, and regulatory acceptance (Ali et al., 2022; Carvalho do Lago et al., 2023).

To address these limitations, a novel class of postbiotic-based formulations derived from *Weissella cibaria* UTNGt21O and *W. confusa* UTNCys2-2 has been developed (Tenea et al., 2025b). The International Scientific Association for Probiotics and Prebiotics defines postbiotics as “preparations of inanimate microorganisms and/or their components that confer a health benefit on the host” (Salminen et al., 2021). They are also described as bioactive products of microbial fermentation, including peptides, enzymes, organic acids, and exopolysaccharides, with antimicrobial and functional properties (Mishra et al., 2024), and more broadly as complex systems of inactivated cells and derived biomolecules capable of modulating microbial ecosystems without requiring viability (Vinderola et al., 2023). In this framework, the formulations composed of peptide–protein (PP) extracts and exopolysaccharides (EPS) are consistent with current definitions and have demonstrated antimicrobial activity *in vitro*, including membrane disruption and reduced bacterial survival on fruit surfaces (Tenea et al., 2025b).

The selection of Weissella strains UTNGt21O and UTNCys2-2 was based on their documented antimicrobial activity, membrane-targeting effects observed by SEM/TEM, and their capacity to produce functional metabolites relevant for postbiotic formulations and biopolymer-based delivery systems (Tenea and Hurtado, 2021). When applied as coatings to strawberries, these formulations delayed spoilage and maintained physicochemical parameters such as firmness, pH, and antioxidant content (Tenea et al., 2025b). In addition, treated fruits showed increased relative abundance of Lactobacillus spp., suggesting a potential role in shaping beneficial microbial communities. However, previous evaluations relied primarily on 16S rRNA amplicon sequencing, which is limited in species-level resolution and does not capture functional or metabolic attributes of the microbiome. As a result, the mechanisms and pathways underlying microbiome responses to these postbiotic formulations remain insufficiently characterized.

To evaluate how postbiotic treatments reshape the strawberry surface microbiota, we performed shotgun metagenomic sequencing on samples treated with three formulations (FF1-FF3) and a commercial disinfectant (CD) after eight days of storage. This approach provided high-resolution insights into both community composition and functional potential. Understanding the effect of the microbiome is crucial for evaluating postbiotic formulations as sustainable postharvest interventions, revealing their capacity to modulate microbial ecosystems, reduce spoilage, and enhance food safety in an ecologically responsible approach. Specifically, we aimed to: (i) resolve treatment-induced shifts in microbial communities at the species level, (ii) annotate functional gene families and their associated metabolic pathways, and (iii) assess the ecological and antimicrobial implications of postbiotic applications through pathway enrichment analyses. By integrating community and functional data, this work provides a systems-level evaluation of postbiotic efficacy as a sustainable postharvest intervention. Our findings advance the understanding of how microbiome-targeted strategies modulate microbial ecosystems on fresh produce, mitigate spoilage, and enhance food safety and shelf life in an ecologically responsible manner.

## 2 Materials and methods

### 2.1 Bacterial strains and antimicrobial formulation preparation

Weissella cibaria UTNGt21O (Gt21O) (GenBank Genome Assembly: SRX8614718) and Weissella confusa UTNCys2-2 (GenBank Accession: KY041684.1) were used to extract precipitated peptide-proteins (PP) and exopolysaccharide (EPS), respectively, as described by Tenea et al. (2025b). In brief, to prepare the PP fraction, the overnight culture of UTNGt21O in MRS broth was centrifuged to yield the cell-free-supernatant (CFS), followed by filtration and precipitation with ethyl acetate (v/v). These precipitates were then dissolved in 25 mM ammonium acetate buffer (pH 6.5), desalted using a laboratory-grade dialysis kit (PURD10005-1KT, Sigma-Aldrich, St. Louis, MO, USA), pre-equilibrated with phosphate buffer (pH 7.0), air-dried in a flow chamber for 48 h, rehydrated with sterile water, and stored at −20 °C. To extract exopolysaccharides (EPS) from *W. confusa* UTNCys2-2, the strain was cultured overnight in MRS broth enriched with 20% sucrose (MRSS) to a final density of approximately 1 × 10^8^ CFU/mL. EPS extraction followed the methodology described by Wang et al. (2023). Three formulations were prepared based on the Minimum Inhibitory Concentration (MIC) of the extracts against a multidrug-resistant *Serratia liquefaciens* P4StpC1 strain, previously isolated from strawberries (Tenea et al., 2025b). The formulations were: FF1 (1 × MIC of PPGt21O combined with EPSCys2-2 at 1:1 v/v), FF2: (1 × MIC of PPGt21O), and FF3: (1 × MIC of EPSCys2-2).

### 2.2 Strawberry sampling and treatment application

Fresh, ripe-stage strawberries (*Fragaria* × *ananassa* L., cv. Monterey) were harvested from an organic commercial farm located in Otavalo, Imbabura Province (Ecuador), a region characterized by volcanic soils (Andisols) in the northern Andes at an average altitude of 2,532 m above sea level, with a temperate climate (average temperatures of 12–18 °C and moderate relative humidity). Fruits were selected based on uniform ripeness (stage four: > 50% red surface area) and absence of physical damage or disease symptoms (López-Valencia et al., 2018). Fruits were surface sanitized by immersion in a 5% sodium hypochlorite solution for 5 min, followed by two rinses with potable water and two with sterile distilled water. Fruits were then air-dried under a laminar flow biosafety cabinet (Class II) for approximately 2 h to minimize external microbial contamination. Strawberries were randomly assigned to four treatment groups: (i) CD (commercial disinfectant), and (ii) FF1, FF2, and FF3 (formulation-treated samples). Each treatment was applied to 100 individual fruits as biological replicates. Treatments were administered by immersing the fruits in the respective solution for 5 min under aseptic conditions, followed by gentle air-drying inside the biosafety cabinet to remove excess liquid. The commercial disinfectant (Star Bac Domestic™, a local bactericidal solution for fruits and vegetables containing citric acid, benzalkonium chloride, glycerol, propylene glycol, glucose, fructose, and water) was prepared according to the manufacturer’s specifications. Following treatment and drying, all samples were placed into sterile plastic trays, sealed, and stored at 4 ± 1°C for eight days under refrigeration to simulate typical postharvest conditions.

### 2.3 Microbial cell collection, DNA extraction and library construction

Microbial cells were detached from the surface of strawberries at eight days of storage using a sterile buffered solution and gentle agitation, followed by filtration and centrifugation to concentrate biomass. Genomic DNA was extracted using the ZymoBIOMICS DNA Miniprep Kit (Zymo Research, #D4300, USA) according to the manufacturer’s protocol. DNA quantity (≥ 12.5 μg/μl), purity (free of RNA/protein), and integrity (main peak > 20 kb) were assessed using a Qubit Fluorometer (Thermo Fisher Scientific). DNA libraries were constructed following standard Illumina protocols (Macrogen, Korea, custom-design sequencing). Genomic DNA was fragmented using a Covaris ultrasonicator to obtain an average insert size of ∼350 bp (PerkinElmer, MA, USA). Fragmented DNA underwent end-repair, A-tailing, and adapter ligation, followed by PCR amplification. Size selection was performed using magnetic beads to obtain fragments in the 200–400 bp range. Library quality and fragment distribution were verified using an Agilent 2100 Bioanalyzer, ensuring appropriate insert size and absence of adapter contamination. Final libraries were quantified prior to sequencing.

Shotgun metagenomic sequencing was performed on an Illumina HiSeq/NovaSeq platform, generating paired-end reads (2 × 150 bp). The workflow is summarized in Supplementary Figure 1.

### 2.4 Metagenomic data processing

Shotgun metagenomic sequencing was performed on each treatment as represented by a single massive composite sample pooled from 100 individual fruits. While standard experimental designs frequently utilize smaller pools with multiple independent sequencing replicates, our massive pooling strategy was a deliberate choice designed to capture a stable population average while mitigating the notoriously high stochastic variability inherent to epiphytic fruit microbiomes. The consistency of the treatment effect and the biological variance across independent replicates were previously established and statistically validated using 16S rRNA amplicon sequencing in a foundational companion study utilizing an identical experimental matrix (Tenea et al., 2025b). Because the biological variation was successfully captured in that prior work, the current shotgun sequencing effort was explicitly designed as a deep-dive, high-resolution functional profiling of those validated population averages. To evaluate the compositional divergence between these pooled samples, we applied a Pearson’s chi-squared test utilizing a simulated distribution based on 10,000 data replicates. While this computational approach cannot replace true biological variance across independent sequencing trials, it allows us to confidently identify robust exploratory trends and structural differences between the deeply sequenced composite communities.

Shotgun metagenomic sequencing data were processed using the KneadData pipeline (v0.10.0)^1^ with the default arguments (Bolger et al., 2014), which integrates Trimmomatic (v0.39) and Bowtie2 (v2.4.5) (Langmead and Salzberg, 2012). Raw reads were demultiplexed into paired-end FASTQ files. Adapter sequences and low-quality bases were removed using Trimmomatic with the parameter SLIDINGWINDOW:4:20, discarding reads with an average Phred quality score < 20 and reads shorter than 50 bp. Shotgun sequencing generated between 38.9 and 41.0 million raw reads per sample across treatments. After quality filtering and host DNA removal, 35.9–37.7 million high-quality reads were retained, corresponding to 91.90–92.68% of the total sequences, indicating high data quality and minimal loss during preprocessing (Supplementary Table 1). Host-derived reads were removed by aligning sequences against the *Fragaria* × *ananassa* reference genome (GCA_034370585.1) using Bowtie2 (v2.4.5), and matching reads were discarded. The resulting high-quality, host-depleted reads were retained for downstream analysis.

Microbial community profiling was performed using MetaPhlAn (v4.0.0), (Blanco-Míguez et al., 2023) operating under default parameters. MetaPhlAn is a computational tool that generates species-level taxonomic profiles of bacteria, archaea, and eukaryotes from shotgun metagenomic data. Taxonomic classification was executed against the MetaPhlAn database release mpa_vOct22_CHOCOPhlAnSGB_202403, which integrates ∼5.1 million clade-specific marker genes derived from approximately 1 million microbial genomes, including both reference genomes (∼236,600) and metagenome-assembled genomes (∼771,500)^2^. This marker-based framework enables unambiguous taxonomic assignment, accurate relative abundance estimation, Species-level Genome Bins (SGB)-level resolution across all microbial domains, strain-level identification and tracking through StrainPhlAn, and large computational speed gains over alignment-based methods, thereby supporting metagenomic strain-level population genomics. Species with < 33% marker gene coverage were considered absent and removed from further analysis. Alpha diversity metrics, including species richness, Shannon, and Gini-Simpson diversity indexes, were calculated from MetaPhlAn-derived species abundance tables using the “calculate_diversity” R script provided within the MetaPhlAn (v4.0.0) package. Beta diversity (weighted and unweighted UniFrac distances) was computed using a pre-calculated phylogenetic tree available in the MetaPhlAn4 database. To test for differences in the community composition resulting from the application of the experimental treatments, we applied a Pearson’s chi-squared test with a simulated distribution based on 10.000 data replicates. The 10 most abundant species were considered for the characterization of community structure across treatments.

### 2.5 Functional profiling and metabolic pathways

Functional profiling and metabolic pathway reconstruction were conducted using HUMAnN v3.5 (Beghini et al., 2021) utilizing default execution parameters. Prior to functional analysis, gene features were filtered to remove taxonomic assignments and uninformative entries, including “UNMAPPED” (reads or gene families that could not be matched to any reference database) and “UNINTEGRATED” (gene families that were successfully identified but could not be assigned to any known metabolic pathway) features. Species-specific gene families were first quantified via nucleotide-level mapping against the ChocoPhlAn pangenome database (mpa_vOct22_CHOCOPhlAnSGB_202403). Unaligned reads were subsequently mapped to the UniRef90 protein database (v2024_06) using DIAMOND blastx for fast and sensitive protein alignment (Buchfink et al., 2015). Functional gene abundances were then assigned based on UniRef90 IDs, and pathway-level abundances were reconstructed using the MetaCyc reference pathway database (Caspi et al., 2020). In addition, EggNOG (Evolutionary Genealogy of Genes: Non-supervised Orthologous Groups) and KEGG (Kyoto Encyclopedia of Genes and Genomes) functional analyses were performed to complement the metabolic profiling. Gene family annotations were assigned using the EggNOG-mapper v2 pipeline, which relies on the EggNOG 5.0 orthology database for high-throughput functional annotation and classification of genes into Clusters of Orthologous Groups (COGs), providing insights into broader functional categories such as metabolism, cellular processes, and information storage (Cantalapiedra et al., 2021). For KEGG analysis, gene sequences were mapped to KEGG Orthology (KO) identifiers using the KEGG Automatic Annotation Server (KAAS), to enable pathway enrichment estimation and to determine the involvement of microbial taxa in specific biochemical routes, including carbohydrate metabolism, secondary metabolite biosynthesis, and antimicrobial compound production (Kanehisa et al., 2016). These additional analyses enabled a comprehensive characterization of the functional potential of microbial communities across treatments. Gene families and metabolic pathways were ranked based on their relative abundance after normalization to copies per million (CPM), and the top 20 features were selected according to their mean abundance across all treatments. Only features consistently detected in at least two samples and exceeding a minimum relative abundance threshold (> 0.01%) were retained for downstream comparative analysis.

## 3 Results and discussion

### 3.1 Community restructuring of the strawberry surface microbiota following postbiotic treatments

**FIGURE 1:**
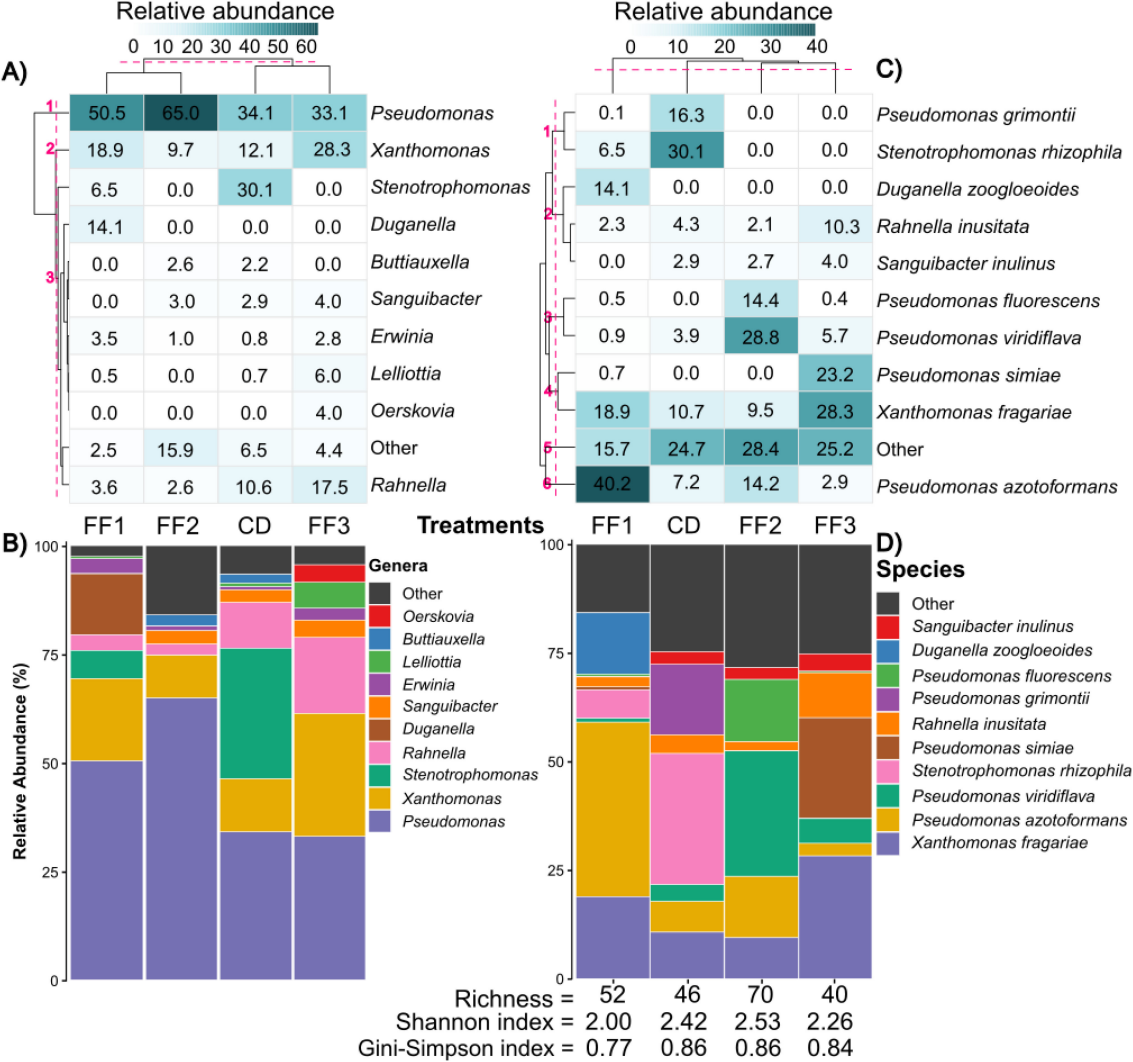
Effects of postbiotic treatments on the composition and abundance of the strawberry surface microbiome. **(A)** Heatmap showing the relative abundance of the most abundant genera across treatments, with hierarchical clustering of samples and taxa. **(B)** Stacked bar plots representing genus-level community composition for each treatment. **(C)** Heatmap of species-level relative abundances with corresponding hierarchical clustering. **(D)** Stacked bar plots showing species-level composition across treatments. Numbers in red identify distinct taxa clusters at the selected distance cut-off value (dotted line). Results of the Pearson’s Chi-square test and diversity indexes are also included. CD: commercial disinfectant; FF1: (1 × MIC, PPGt21O + EPSCys2-2: 1:1, v/v), FF2: (PPGt21O, 1 × MIC), FF3: (EPSCys2-2, 1 × MIC).

Shotgun metagenomic profiling revealed robust exploratory trends indicating treatment-dependent restructuring of the strawberry surface microbiota (Figure 1). Compositional comparisons of the pooled samples demonstrated distinct structural shifts at both the genus (χ^2^ = 395.38, df = NA, *p*-value = 0.0001, replicates = 1,000) and species levels (χ^2^ = 203.95, df = NA, *p*-value = 0.0001, replicates = 1,000). While the core microbiome was consistently dominated by *Pseudomonas* across all groups, the specific postbiotic formulations (FF1, FF2, FF3) drove distinct shifts in community composition compared to the control (CD) (Figures 1A,B). *Pseudomonas* was the most abundant genus in all treatments but exhibited marked variation in relative abundance, peaking in FF2 (64.99%) and FF1 (50.48%) compared to CD (34.14%) and FF3 (33.06%). This suggests that FF1 and FF2 formulations may exert selective pressure that favors *Pseudomonas* proliferation or suppresses competing taxa. In contrast, FF3 maintained *Pseudomonas* levels comparable to the control, indicating a more moderate impact on this dominant genus (Figures 1A,B).

Hierarchical clustering resolved two primary groups among the treatments: a CD-FF3 cluster and an FF1-FF2 cluster for the estimated abundances at the genus level (Figure 1A). The CD-FF3 grouping was driven by shared compositional features, specifically the retention of moderate *Pseudomonas* levels. However, a major divergence was observed in *Stenotrophomonas*. While *Stenotrophomonas* was a co-dominant genus in the control (30.09%), it was strongly suppressed in FF3 (0.00%) and FF2 (0.02%), suggesting that postbiotic treatments effectively eliminate this sanitizer-tolerant genus. The FF1-FF2 cluster of treatments diverged most from the control. FF2 was characterized by a distinct loss of evenness, becoming heavily dominated by *Pseudomonas* at the expense of other genera. FF1, while also high in *Pseudomonas*, was uniquely distinguished by a bloom of *Duganella* (14.14%), a genus virtually absent in all other groups (Figures 1A,B).

Clustering of the ten most abundant genera defined three main ecological groups. (1) The first cluster consisted solely of *Pseudomonas*, which functioned as a hyper-dominant generalist across all treatments, with marked expansion under FF1 and FF2, likely displacing other taxa. (2) The second cluster comprised *Xanthomonas* as a single-member group, representing a persistent co-dominant taxon (Alvarez-Martinez et al., 2020). It remained highly abundant in FF3 (28.29%), likely occupying the niche vacated by *Stenotrophomonas*, and decreased in FF2 (9.74%) where *Pseudomonas* dominance was strongest. *Xanthomonas* persistence across treatments, including FF1, is consistent with adaptive traits such as biofilm formation, extracellular polysaccharide production, and stress tolerance, which can reduce the penetration and activity of membrane-targeting postbiotic compounds, indicating a selective rather than complete antimicrobial effect. (3) The remaining genera formed a third cluster, comprising taxa that responded to specific treatments. This group included potentially beneficial genera such as *Rahnella* and *Lelliottia*, co-enriched in FF3 (17.51% and 5.96%, respectively), suggesting the establishment of a more balanced consortium. In contrast, transient taxa such as *Duganella* (exclusive to FF1) and *Oerskovia* (exclusive to FF3) further reflect formulation-specific microbiome restructuring.

These data indicate that while all postbiotic treatments altered the microbiome, they did so through distinct ecological trajectories: FF2 drove a simplification favoring *Pseudomonas* dominance; FF1 selected for unique taxa like *Duganella*; and FF3 promoted a diverse, potentially beneficial consortium (*Rahnella* and *Lelliottia*) while successfully suppressing the control-associated *Stenotrophomonas*.

Metagenomic profiling at the species level resolved the specific ecological drivers behind the community shifts (Figures 1C,D), revealing that broadly defined genus-level changes were driven by the selective enrichment of distinct species guilds. The control treatment (CD) was characterized by the dominance of *Stenotrophomonas rhizophila* (30.09%) and *Pseudomonas grimontii* (16.27%). The persistence of these taxa suggests they possess intrinsic tolerance to standard washing procedures. Notably, postbiotic treatments proved highly effective against this specific guild: *P. grimontii* was virtually eliminated in all treatments (< 1%), and *S. rhizophila* was reduced to 6.49% in FF1 and eradicated in FF2 and FF3.

Each postbiotic treatment selected for a unique set of dominant species, confirming distinct modes of action (Figures 1C,D): (1) The FF1 formulation drove a massive expansion of *Pseudomonas* azotoformans (40.21%), which replaced *P. grimontii* as the dominant pseudomonad. Uniquely, FF1 enriched *Duganella zoogloeoides* (14.14%), a species virtually absent in all other groups. Members of the genus *Duganella* are commonly associated with soil and plant environments and have been reported to exhibit metabolic versatility, including the ability to utilize diverse carbon substrates and produce bioactive secondary metabolites that may contribute to microbial competition and ecological adaptation (Zhao et al., 2022). Their enrichment in FF1 suggests that this formulation may create a selective niche favoring stress-tolerant, metabolically flexible taxa capable of exploiting postbiotic-derived substrates or occupying ecological space vacated by suppressed competitors. (2) For the FF2 formulation, niche space vacated by the elimination of *S. rhizophila* was occupied by a diverse guild of fluorescent pseudomonads. Dominance was shared between *P. viridiflava* (28.85%), *P. fluorescens* (14.39%), and *P. azotoformans* (14.17%). This indicates that FF2 favors several generalists of rapid colonizers rather than a single specialist. (3) FF3 established the most ecologically distinct profile. It was dominated by *Xanthomonas fragariae* (28.29%), but unlike other treatments, this was coupled with the strong co-enrichment of *P. simiae* (23.24%) and multiple *Rahnella* species (*R. inusitata* and *R. aquatilis*, combined ∼17%). The presence of *Rahnella* and *Lelliottia* amnigena (5.96%), taxa associated with plant growth promotion, suggests FF3 supports a functional, multi-species guild.

Hierarchical clustering based on abundance patterns identified five distinct groups of species, which mapped to specific treatment conditions (Figure 1C). (1) A first cluster formed by *S. rhizophila* and *P. grimontii* defined the microbiome of the control fruit (CD). *S. rhizophila* was the most abundant species in the control (30.09%), co-occurring with *P. grimontii* (16.27%). A consistent pattern across all postbiotic treatments (FF1, FF2, FF3) was the destabilization of this cluster; *P. grimontii* was reduced to < 1% in all treated groups, and *S. rhizophila* was largely eliminated, particularly in FF2 and FF3. (2) The second cluster comprised species that showed high specificity to single treatments. *Duganella zoogloeoides* was found exclusively in FF1 (14.14%), acting as a unique marker for this formulation. In contrast, *R. inusitata* was most prominent in FF3 (10.33%), while *Sanguibacter inulinus* maintained a low but consistent presence across most groups. (3) FF2 was characterized by the enrichment of Cluster 3. Unlike the single-species dominance seen in FF1, FF2 promoted a co-occurrence of *P. viridiflava* (28.85%) and *P. fluorescens* (14.39%). While *P. viridiflava* was present in other groups, its peak abundance, combined with the highest levels of *P. fluorescens*, distinguished the FF2 community structure. (4) Cluster 4 defined the specific profile of FF3. This treatment was unique in enriching *Pseudomonas simiae* (23.24%), a species absent or negligible in the other groups. This cluster also included *X. fragariae*, which, while present across all treatments, reached its highest relative abundance in FF3 (28.29%). (5) The restructuring in FF1 was primarily driven by Cluster 5. *P. azotoformans* became the hyper-dominant species in this treatment (40.21%), contrasting with its lower abundance in the control (7.17%) and FF3 (2.91%). This species clustered with the “Other” category, which likely reflects the low-abundance diversity remaining in the background of this treatment.

Overall, the clustering analysis indicates that the postbiotic treatments shifted the community away from the *S. rhizophila*/*P. grimontii* baseline (Cluster 1) toward distinct alternative states: FF1 favored *P. azotoformans* (Cluster 5) and *D. zoogloeoides* (Cluster 2); FF2 favored a *P. viridiflava*/*P. fluorescens* guild (Cluster 3); and FF3 selected for *P. simiae* and *X. fragariae* (Cluster 4). Our previous 16S rRNA amplicon survey provided preliminary insight into treatment-driven microbial shifts but was limited by genus-level resolution and the inability to distinguish closely related species (Tenea et al., 2025b). As a result, key taxa such as *P. simiae*, *X. fragariae*, or *R. victoriana* were not detected. In contrast, shotgun metagenomics enabled high-resolution, species-level identification and functional insight, revealing shifts that were undetectable by amplicon sequencing, including the suppression of specific pathogens by FF2 and the enrichment of functionally beneficial taxa. These findings align with recent reports demonstrating antimicrobial and microbiota-modulating properties of LAB-derived postbiotics (García-Gamboa et al., 2024; Tenea et al., 2025b), supporting the potential of FF1 and FF2 as sustainable postharvest interventions. Future work should assess functional outcomes over storage time and across cultivars to evaluate their performance under commercial conditions.

### 3.2 Bacterial roles, richness, and diversity across treatments

The alpha-diversity measures provide insight into the antimicrobial effects of the postbiotic formulations compared with the commercial disinfectant (Figure 1D). FF1, despite slightly increasing richness relative to CD (52 vs. 46 species), exhibited the lowest Shannon (2.00) and Gini-Simpson (0.774) indices, indicating a community where a small group of taxa are dominant in abundance. This pattern is consistent with a broad-spectrum antimicrobial effect, in which FF1 suppresses potentially pathogenic species (e.g., *P. viridiflava* and *P. fluorescens*) (Table 1), but allows the proliferation of *P. azotoformans*, known as a plant growth-promoting species (Schober et al., 2024; Bonotto et al., 2025). However, the abundance of *X. fragariae*, a well-known deleterious species for Strawberry production (Wei et al., 2024; Ma et al., 2025), is second to all treatments after FF3 (Table 1). We propose that an uneven community (i.e., low diversity) is the result of a strong inhibitory pressure, this supports FF1 as the treatment with the most pronounced antimicrobial action. In contrast, FF2 produced the highest richness (70 species) and the highest Shannon (2.53) and Gini-Simpson (0.861) indices, reflecting a diverse, more evenly structured microbiota, and therefore a non-selective inhibitory effect (Figure 1D). However, this group was dominated by the phytopathogen *P. viridiflava* (28.8% relative abundance) and the spoilage-associated *P. fluorescens* (14.4%) (Córdova et al., 2022); yet, notably, with the lowest abundance for the phytopathogen *X. fragariae* (Table 1).

**TABLE 1:** Risk assessment of the 10 most abundant bacterial species according to BacDive.

Species	Pathogenicity plant	Pathogenicity animal	Pathogenicity human	Biosafety level
*Sanguibacter inulinus*	–	Yes, in single cases		1
*Duganella zoogloeoides*	–	–	–	1
*Pseudomonas fluorescens*	–	Yes	Yes, in single cases	1
*Pseudomonas grimontii*	–	–	–	1
*Rahnella inusitata*	–	–	–	1
*Pseudomonas simiae*	–	Yes	–	2
*Stenotrophomonas rhizophila*	–	–	–	1
*Pseudomonas viridiflava*	Yes	–	–	1
*Pseudomonas azotoformans*	–	–	–	1
*Xanthomonas fragariae*	Yes	–	–	1

Interaction and safety attributes provided by BacDive on the assessed species of bacteria. Biosafety levels used by BacDive are according to the four-level classification by the Federal Institute for Occupational Safety and Health at Germany: level 1 = Biological agents that are unlikely to cause human disease; level 2 = Biological agents that can cause human disease and might be a hazard to employees; they are unlikely to spread to the community; there is usually effective prophylaxis or treatment available; level 3 = Biological agents that can cause severe human disease and present a serious hazard to employees; they may present a risk of spreading to the community, but there is usually effective prophylaxis or treatment available; level 4 = Biological agents that cause severe human disease and are a serious hazard to employees; they may present a high risk of spreading to the community; there is usually no effective prophylaxis or treatment available.

FF3 exhibited the lowest richness (40 species) and intermediate diversity values, implying strong and non-selective antimicrobial impact (Figure 1D). Yet, the large abundance for *X. fragariae* as a phytopathogen is considered problematic. The second most abundant species in this group is *Pseudomonas simiae*, which is a well-characterized plant growth-promoting rhizobacterium (Desrut et al., 2020) but is also the only abundant species with biosafety level 2 (Table 1). The CD treatment is characterized by the noticeable abundance of *Stenotrophomonas rhizophila* and *P. grimontii* (Figure 1D). The first is non-pathogenic, a promoter of plant growth, and a pathogen inhibitor (Raio et al., 2023: Pinski et al., 2020). The second has been associated with turnip root rot (Sawada et al., 2019) but is capable of biofilm production with potentials for biocontrol (Sprotte et al., 2021; Alymanesh and Ghanbari, 2025).

In contrast to the previous 16S rRNA amplicon analysis (Tenea et al., 2025b), which included baseline comparisons and indicated relative stability in alpha diversity while capturing only broad genus-level patterns, the present shotgun metagenomic approach provides species-level resolution and functional insight, enabling a more detailed characterization of treatment-driven microbiome restructuring. This higher resolution reveals distinct ecological trajectories across formulations, including the dominance of *P. azotoformans* in FF1, the enrichment of fluorescent *Pseudomonas* species (e.g., *P. viridiflava* and *P. fluorescens*) in FF2, and the prevalence of *X. fragariae* in FF3, together with pathway-level changes that support a more mechanistic interpretation of postbiotic–microbiome interactions beyond amplicon-based approaches. However, it is important to distinguish between taxonomic and functional baselines. Although the initial community composition was previously characterized using 16S rRNA sequencing (Tenea et al., 2025b), our approach for the present study did not capture functional gene content. Because we did not measure the metagenome at the start of the experiment, the functional baseline remains unresolved in the present study. We also did not include a blank control (e.g., water-washed samples evaluated at Day 8), which limits direct assessment of the natural trajectory of the microbiome during storage. Therefore, the observed taxonomic and functional shifts are interpreted relative to the commercial disinfectant (CD) reference rather than as absolute changes from an initial state of the metagenome.

Besides, the reduced antimicrobial efficacy observed in FF2 and FF3 can be explained by the biochemical composition of each formulation: while the PP extract (PPGt21O) contains bacteriolytic components capable of disrupting membrane integrity, the EPS fraction (Cys2-2) is a complex polysaccharide matrix enriched in hydroxylated carbohydrates, uronic acids, and minor protein and organic acid residues, as indicated by previous FTIR and ^1^H-NMR analyses (Ascanta et al., 2025). This composition confers high viscosity, water retention capacity, and abundant functional groups (−OH, −COO^–^), which can promote biofilm-like structuring, limit antimicrobial diffusion, and facilitate microbial adhesion and persistence in the absence of active antimicrobial compounds, thereby reducing overall inhibitory efficacy when EPS is applied alone (FF3) or without optimized matrix-assisted delivery (FF2).

In summary, the shifts in alpha diversity and taxonomic composition indicate that the postbiotic formulations modulate the strawberry surface microbiome differently relative to the commercial disinfectant (CD). The CD treatment maintained a community associated with *Stenotrophomonas rhizophila*, whereas FF1 showed the most consistent antimicrobial profile. The lower diversity indices in FF1 are consistent with a selective pressure reflected in the reduced relative abundance of key phytopathogens such as *P. viridiflava* and *P. fluorescens*, together with the increased abundance of *P. azotoformans*. In contrast, the higher diversity observed in FF2 and the pathogen-associated composition in FF3 indicate a less selective effect, allowing the persistence of deleterious taxa. Overall, FF1 presents the most suitable biocontrol profile among the tested formulations, although further adjustment is needed to address persistent taxa such as *X. fragariae*. Accordingly, the diversity patterns described here represent comparative outcomes relative to the CD baseline and do not fully capture microbiome dynamics under untreated storage conditions.

### 3.3 Beta-diversity analysis between samples

**FIGURE 2:**
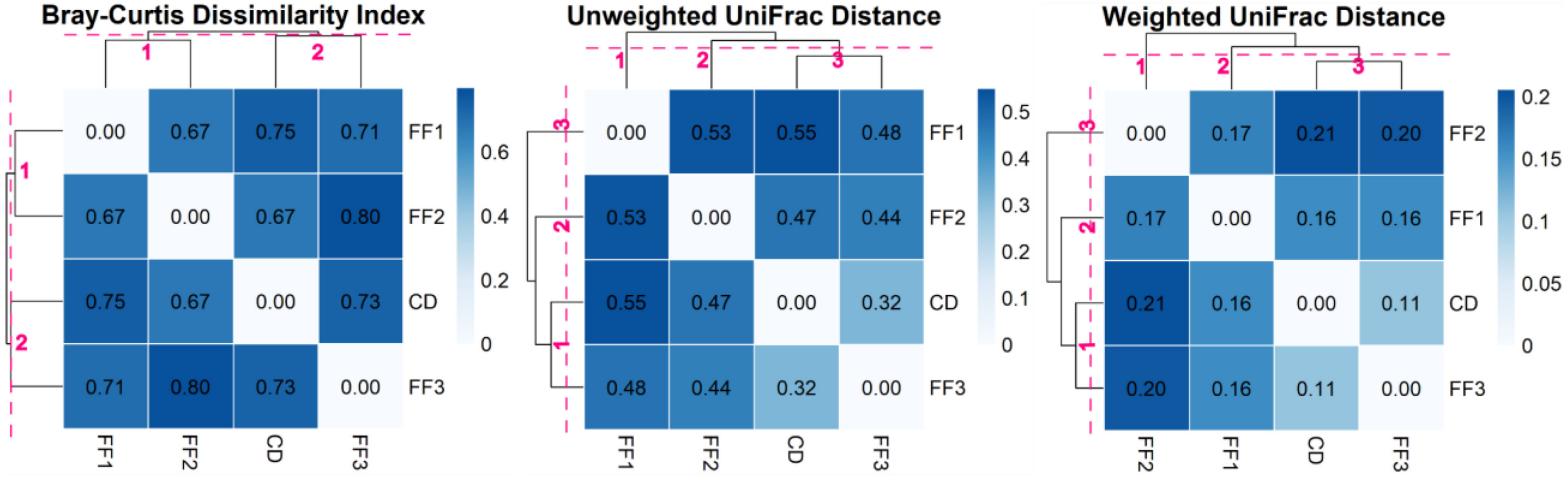
Heatmaps of beta diversity distance showing microbial composition dissimilarity between samples. Color intensity indicates the magnitude of dissimilarity. Numbers in red identify distinct treatment clusters at the selected distance cut-off value (dotted line).

Beta-diversity analyses demonstrated clear treatment-specific restructuring of the strawberry surface microbiota (Figure 2). Evaluation of abundance-based compositional differences via the Bray-Curtis dissimilarity index revealed that FF1 exhibited the greatest divergence from CD (0.753), followed by FF3 (0.735) and FF2 (0.674). Notably, the highest overall dissimilarity within the matrix occurred between FF2 and FF3 (0.80), indicating that the individual postbiotic components drive entirely distinct microbial profiles (Figure 2). Presence/absence–based dissimilarity (unweighted UniFrac distances) further revealed that FF3 retained a community most like CD (0.321), whereas formulations containing the peptide-protein extract (FF1 and FF2) induced far greater taxonomic turnover (0.548 and 0.471, respectively). Weighted UniFrac distances corroborated these patterns, showing minimal phylogenetic divergence between CD and FF3 (0.108) but more pronounced abundance-weighted shifts under FF1 (0.157) and FF2 (0.205) (Figure 2). The hierarchical clustering of these metrics visually confirms these trajectories. In both phylogenetic analyses, CD and FF3 consistently cluster together into a single clade, whereas FF1 and FF2 drive the microbiome into a structurally distinct alternative state.

However, the moderate phylogenetic divergence between CD and FF3 suggests that the exopolysaccharide carrier alone exerts a selective pressure like that of standard chemical sanitizers, which are known to reduce overall load while leaving behind a phylogenetically similar baseline of tolerant populations. In contrast, the pronounced compositional and phylogenetic shifts induced by FF1 and FF2 indicate that the peptide-protein (PP) extract is the primary driver of deep microbial restructuring, forcing the community out of its conventional stress-adapted state and establishing a novel ecological equilibrium (Figure 2).

### 3.4 Functional modulation of fruit surface microbiota by postbiotic treatments

**FIGURE 3:**
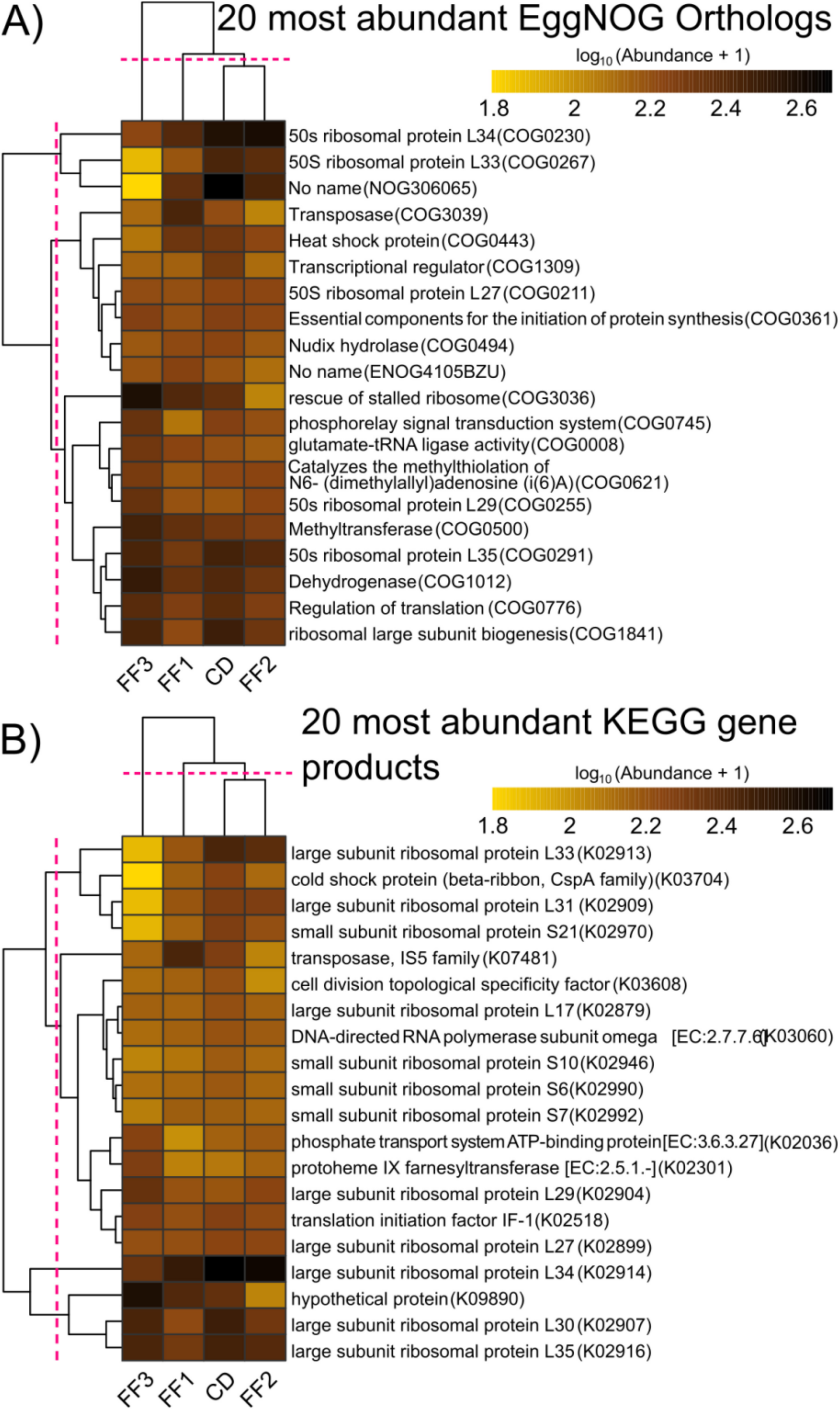
Functional gene abundance, EggNOG **(A)** and KEGG **(B)** annotation across treatments. Color intensity represents log-transformed gene abundance (Log10[Abundance + 1]), with darker shades indicating higher abundance levels. Hierarchical clustering highlights grouping of gene families with similar treatment-specific expression patterns. CD-commercial disinfectant; FF1: (1 × MIC, PPGt21O + EPSCys2-2: 1:1, v/v), FF2: (PPGt21O, 1 × MIC), FF3: (EPSCys2-2, 1 × MIC). The red dotted line represents the selected distance cut-off value to establish clusters.

To understand the metabolic implications of the microbial compositional shifts induced by the postbiotic formulations, we evaluated the functional potential of the strawberry surface microbiome. The hierarchical clustering of these functional profiles (Figure 3) revealed distinct metabolic signatures associated with each treatment, corroborating the taxonomic variations previously observed; it resolved three primary groups among the treatments: a CD-FF2 cluster and exclusive clusters for FF1 and FF3. The analysis of eggNOG orthologous groups (Figure 3A) demonstrated a clear functional divergence between the postbiotic treatments and the commercial disinfectant (CD). The FF1 treatment, which exhibited the most robust antimicrobial profile taxonomically, was characterized by a marked reduction in the relative abundance of core metabolic and replicative functions. For instance, specific structural components of the translation machinery, including 50S ribosomal proteins L34 (COG0230) and L27 (COG0211), showed lower relative abundances in FF1 compared to the CD and FF2 treatments. This reduction in the relative abundance of ribosomal protein genes suggests a potential limitation in the functional capacity for protein synthesis and is consistent with a constrained microbial growth potential in the FF1-treated microbiome. In addition, functional categories associated with environmental stress adaptation were maintained or slightly enriched in the FF1-treated microbiome. The abundance of heat shock proteins (COG0443) remained high in FF1, suggesting that the surviving microbiota, such as the resilient *Stenotrophomonas rhizophila* populations identified in earlier sections, relies on elevated stress-response mechanisms to persist in the presence of the postbiotic antimicrobial compounds. Besides, fluctuations in methyltransferase activity (COG0500) across treatments highlight potential shifts in regulatory and epigenetic mechanisms as the microbial community adapts to the distinct biochemical environments introduced by the formulations. These functional patterns may help explaining the taxonomic shifts observed in FF1 and FF2, particularly the increased dominance of *Pseudomonas*. In FF2, and to a lesser extent in FF1, functional profiles indicated enrichment of pathways related to carbohydrate metabolism, amino acid biosynthesis, and stress tolerance, which are characteristic of metabolically versatile and fast-growing taxa such as *Pseudomonas* spp. This suggests that postbiotic-derived compounds may selectively favor organisms capable of rapid resource utilization and adaptive stress responses. In parallel, the suppression of competing taxa, such as *Stenotrophomonas* and other less abundant genera, likely reduced niche competition, further facilitating *Pseudomonas* expansion. Together, these findings indicate that the proliferation of *Pseudomonas* in FF1 and FF2 is driven by functional adaptation to the altered metabolic landscape imposed by postbiotic treatments, rather than stochastic variation.

The shifts observed in the COG categories were further supported by the KEGG orthologs profiling (Figure 3B), which provided a higher-resolution view of specific metabolic pathways. The hierarchical clustering of KEGG orthologs confirmed that the functional capacity of the microbiome was notably restructured, particularly under the FF1 and FF3 treatments. In alignment with the eggNOG results, key KEGG orthologs integral to the translation machinery, such as the large subunit ribosomal proteins L34 (K02914), L33 (K02913), and L35 (K02916), alongside small subunit components (K02946), were notably reduced in abundance in the FF1 treatment relative to the CD control (Figure 3B). This reinforces the hypothesis that the FF1 postbiotic formulation establishes a suppressive environment that actively curtails the basic replicative capacity of spoilage-associated bacteria. Furthermore, metabolic transport systems were fundamentally altered. The relative abundance of the phosphate transport system ATP-binding protein (K02036) was notably reduced in FF1. A decrease in the relative abundance of genes associated with nutrient acquisition pathways suggests a potential reduction in the functional capacity for resource uptake and is consistent with a more constrained metabolic environment for the epiphytic microbiome, which may limit the proliferation of opportunistic pathogens and spoilage organisms.

**FIGURE 4:**
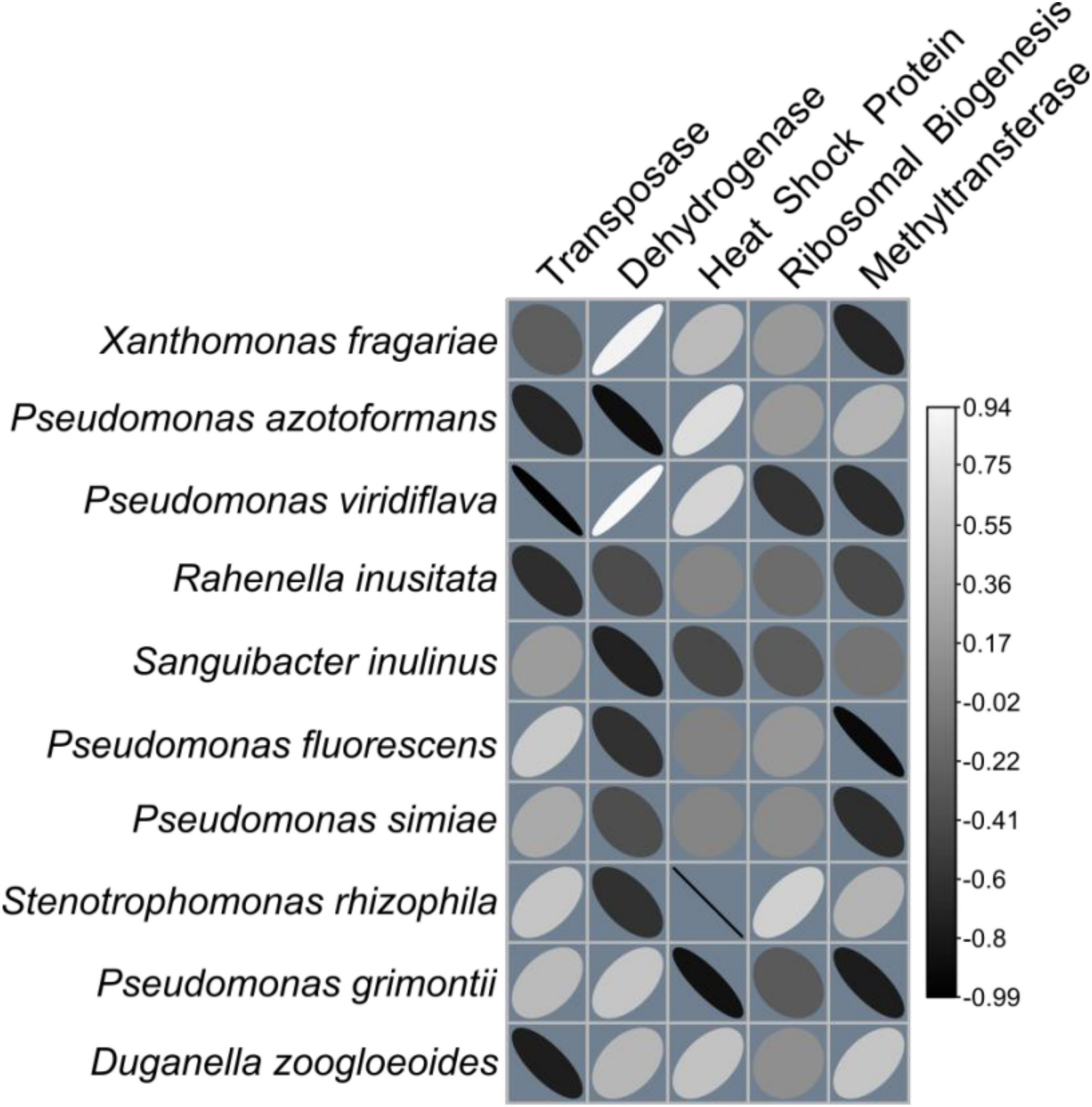
Correlogram exploring abundance relationships between the 10 most abundant bacterial species and the 5 most abundant functional gene categories (e.g., ribosomal proteins, methyltransferases, transposases). Pearson correlation coefficients (r) were calculated across four datapoints which are the pooled treatment conditions (*n* = 4: CD, FF1, FF2, FF3). Due to the very limited sample size (*n* = 4), these correlations should be interpreted strictly as exploratory trends and not as statistically robust associations. Ellipses oriented to the right (white) indicate positive relationships, whereas ellipses oriented to the left (black) indicate negative relationships.

In contrast, the functional profile of the FF3 treatment, which taxonomically permitted the enrichment of phytopathogens like *Xanthomonas fragariae*, exhibited a distinct metabolic signature. While it also showed some reduction in certain ribosomal proteins compared to the CD, the overall functional clustering suggests a different compensatory metabolic state, likely driven by the blooming opportunistic taxa utilizing alternate survival pathways. Considering the measured abundance for KEGG orthologs of relevance to translation, such as 50s ribosomal protein L35 (COG0291), regulating protein (COG0776), and ribosomal large subunit biogenesis (COG1841), the FF1 treatment maintains a comparative advantage over the FF3 treatment as an overall inhibitor of this key bacterial metabolism (Figure 3B). However, these functional profiles demonstrate that the application of LAB-derived postbiotics, specifically the FF1 formulation, does not merely alter the superficial taxonomic composition of the strawberry surface. Instead, it fundamentally reshapes the metabolic landscape, shifting the microbial community from an active, growth-oriented state toward a dormant or stress-adapted state, thereby exerting a protective, life-extending effect on the postharvest fruit. To further elucidate how the postbiotic formulations reshape the surface microbiome, we evaluated the co-occurrence patterns between the relative abundances of key taxa and major functional gene categories (Figure 4). It is important to note that in bulk metagenomic profiles, a strong correlation does not strictly assign a specific gene to a particular taxon; rather, it highlights ecological synchronization. It reveals how specific community assemblages, driven by the selective pressures of the postbiotic treatments, co-vary with distinct metabolic states.

To further visualize potential co-occurrence trends between community structure and metabolic potential across the evaluated conditions, we computed Pearson correlation coefficients between the relative abundances of key taxa and major functional gene categories across the four pooled treatment groups (*n* = 4) (Figure 4). Given that these correlations are derived from only four pooled data points (*n* = 4), they should be interpreted as hypothesis-generating patterns of ecological co-variation rather than evidence of mechanistic relationships and require future validation with larger sample sizes to establish robust mechanistic causality. Nevertheless, this exploratory analysis highlighted distinct compositional alignments that differentiate the treatment environments. Notably, opportunistic bacteria that expanded in the less restrictive treatments, such as *P. viridiflava* (enriched in FF2) and the phytopathogen *X. fragariae* (enriched in FF3), showed strong positive abundance alignments with dehydrogenase activity (*r* = 0.94 and *r* = 0.90, respectively) (Figures 1, 4). Dehydrogenases are critical drivers of oxidative metabolism and energy production (Quijano et al., 2016). The synchronization between these taxa and dehydrogenase abundance suggests a potential ecological trend where postbiotic formulations (like FF2 and FF3) fail to fully suppress these opportunists; thus, the overall community shifts toward a highly active, energy-consuming metabolic state characteristic of early fruit spoilage.

Conversely *S. rhizophila*, which was dominant under the commercial disinfectant (CD) but severely depleted by the postbiotics (FF1-FF3), exhibited a strong inverse abundance trend with Heat Shock Proteins across the four sample pools (*r* = −0.99) (Figures 1, 4). This suggests an overarching trend where the application of postbiotic formulations corresponds with both the structural depletion of *S. rhizophila* populations and a simultaneous community-wide increase in stress-response mechanisms (HSPs). Ultimately, these exploratory co-occurrence patterns illustrate that the dismantling of the active baseline community aligns closely with the shift toward a highly stressed functional landscape, particularly under the FF1 formulation.

Furthermore, strong negative correlations were observed between dominant spoilage organisms and specific regulatory functions. For instance, *P. fluorescens* negatively correlated with methyltransferase activity (*r* = −0.91) (Figure 4), suggesting that the proliferation of such proteolysis-driven spoilage bacteria is associated with a community-wide reduction in abundance of these specific epigenetic or regulatory pathways. Ultimately, these co-occurrence patterns demonstrate that the postbiotics, particularly the highly suppressive FF1 formulation, do not act randomly. They systematically dismantle the active, oxidative community state associated with opportunists like *P. viridiflava*, replacing it with a suppressed, highly stressed microbiome landscape.

### 3.5 Metabolic pathway modulation across treatments

**FIGURE 5:**
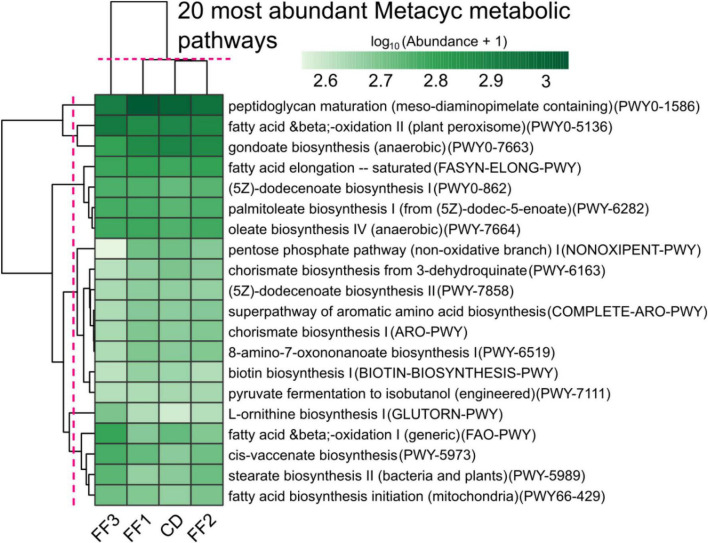
Heatmap of MetaCyc metabolic pathway abundance identified in cell-free supernatants from the commercial disinfectant (CD) and formulations (FF1, FF2, FF3). The color scale represents the log10-transformed abundance values (log10[Abundance + 1]) of pathway-associated metabolites. Hierarchical clustering reveals differential pathway enrichment among samples, with notable variation in fatty acid metabolism, peptidoglycan maturation, and amino acid biosynthesis pathways. The red dotted line represents the selected distance cut-off value to establish clusters.

To gain a systems-level understanding of how the postbiotic formulations influence the functional trajectory of the strawberry surface microbiome, we reconstructed the metabolic pathways using the MetaCyc database. The first 20 most abundant profiles of these key metabolic pathways, derived from the metagenomic data (Figure 5), reveal profound shifts in the community’s energetic and biosynthetic capacities, particularly concerning lipid metabolism and structural biosynthesis, depending heavily on the applied treatment. The hierarchical clustering of the treatments in Figure 5 underscores this functional divergence. Notably, the highly suppressive FF1 formulation segregates distinctly from the less restrictive treatments (FF2 and FF3), which group together driven by their shared signatures of active metabolic proliferation and lipid degradation.

#### 3.5.1 Targeted suppression of lipid degradation and membrane biosynthesis by FF1 formulation

The MetaCyc profile of the FF1 treatment illustrates a targeted metabolic bottleneck that aligns with the previously discussed taxonomic reduction of spoilage organisms. Rather than a universal silencing of all biological activity, FF1 restricts specific pathways associated with active microbial proliferation and host tissue degradation. Most notably, the abundance data demonstrates that fatty acid ß-oxidation I (generic) and fatty acid ß-oxidation II (plant peroxisome) were suppressed to their lowest levels under the FF1 treatment compared to the other formulations. Because opportunistic fruit pathogens often utilize ß-oxidation to rapidly metabolize host lipids during the initial stages of spoilage, the reduction in abundance of these specific degradation pathways suggests that FF1 effectively arrests the enzymatic breakdown of the fruit’s protective outer layers. Furthermore, FF1 effectively curtailed the biosynthesis of essential cellular structural components. The pathway governing stearate biosynthesis II (bacteria and plants) exhibited its lowest relative abundance under this formulation. The suppression of this specific lipid biosynthesis pathway indicates that the postbiotic paralyzes the ability of the surviving bacterial community to synthesize new cell membranes, thereby halting active colonization and population expansion. However, the pathway for peptidoglycan maturation (meso-diaminopimelate containing) reached its highest overall relative abundance in the FF1 treatment. We propose that while the surviving microbiota are not actively dividing, they are heavily enriched in cell-wall reinforcement mechanisms as a structural defense strategy against the harsh antimicrobial environment. Interestingly, specific core survival and regulatory pathways, such as biotin biosynthesis I and chorismate biosynthesis I, maintained stable or slightly elevated abundances in FF1. This differential regulation suggests that the FF1 environment forces the community into a highly restricted, survival-oriented metabolic state dominated by a few tolerant taxa, rather than indiscriminately eradicating the microbiome like a broad-spectrum chemical bactericide.

#### 3.5.2 Metabolic proliferation and spoilage signatures in FF3

In contrast, treatment FF3 exhibited a highly active metabolic signature that could be interpreted as an indicative of active spoilage. The data shows a distinct enrichment in both energy-yielding and structural pathways in this treatment. The fatty acid ß-oxidation I (generic) and fatty acid ß-oxidation II (plant peroxisome) pathways that were heavily suppressed in FF1 reached their highest relative abundances under the FF3 treatment. This strong enrichment in abundance directly correlates with the blooming of opportunistic taxa, reflecting an active, energy-consuming metabolic state driven by the breakdown of complex plant lipids (Zaman et al., 2025). Similarly, membrane synthesis pathways, including cis-vaccenate biosynthesis, stearate biosynthesis II (bacteria and plants), and fatty acid elongation, peaked in the FF3 treatment. The enrichment of these specific biosynthetic pathways mirrors the active cell division and rapid population growth of the spoilage organisms permitted by this less restrictive formulation. The CD and FF2 treatments are a stress-adapted baseline, maintaining relatively high levels of baseline peptidoglycan maturation and specific fatty acid biosynthesis (e.g., gondoate biosynthesis, PWY-7663), which represents a conventional survival response to chemical sanitizers. Collectively, the pathway reconstruction analysis confirms that the superior preservative effect of the FF1 formulation stems from its targeted ability to restrict specific fatty acid metabolism networks. We propose that by simultaneously preventing the breakdown of host lipids via ß-oxidation and halting bacterial membrane synthesis, FF1 forces the fruit surface microbiome into a state of metabolic dormancy, preventing the onset of macroscopic spoilage.

## 4 Conclusion

Our exploratory study suggests that LAB-derived postbiotic formulations can reshape the structure and predicted functional potential of the strawberry surface microbiome under controlled laboratory conditions. Using an exploratory integrated metagenomic approach, the FF1 formulation was associated with the most pronounced ecological shift, moving the epiphytic community away from an active, spoilage-associated state toward a profile consistent with reduced metabolic activity. Functional pathway analysis suggested a potential dual mode of action, involving suppression of microbial growth and spoilage-associated metabolism, reflected by a lower relative abundance of genes linked to translation and lipid metabolism, alongside a relative increase in stress-response functions within the residual microbiota. In contrast, our results suggest that FF2 and FF3 promoted microbial profiles associated with opportunistic taxa and higher metabolic activity. These observations highlight the potential of LAB-derived postbiotics to modulate fruit-associated microbiomes at both taxonomic and functional levels. However, given that functional inferences are derived from gene abundance profiles and supported by exploratory statistical approaches with limited replicates, these findings should be interpreted as predictive rather than definitive evidence of metabolic activity. Further fine-scale structural characterization is an ongoing target for separate biochemical research that can determine the molecular nature of our postbiotic formulations. However, given that this study was conducted under laboratory conditions, further validation under field and commercial postharvest environments is also necessary to confirm the efficacy of the formulations, their scalability, their effect on commercial fruit quality, and their application to practical commercial scenarios, as sustainable alternatives to conventional disinfectants. While the use of massive composite sampling (pooling 100 fruits per treatment) presents an experimental limitation by restricting replicate-level resolution within the shotgun dataset, this approach provided a crucial analytical strength. By mitigating localized, individual-level stochastic noise, this sampling strategy enabled the deep sequencing of stable population averages, allowing us to identify robust exploratory trends and potential ecological and functional shifts occurring across the treated microbial populations.

Finally, while this study provides high-resolution insights into the bacterial metabolic response to LAB-derived postbiotics, we acknowledge that the strawberry surface is a multi-kingdom ecosystem. Postharvest spoilage is heavily driven by fungal pathogens, and our previous work mapping the strawberry mycobiome (Tenea et al., 2024) highlights the complexity of these eukaryotic communities. Because the current study focused specifically on bacterial functional pathways and metabolic dormancy, evaluating the cross-domain interactions, specifically how postbiotic-induced bacterial dormancy influences fungal survival and proliferation, remains a critical limitation of this single-kingdom approach. Future shotgun metagenomic efforts must aim to capture the complete bacterial-fungal interactome to fully predict the ecological outcomes of postbiotic preservation strategies.

## Data Availability

The original contributions presented in this study are included in this article/Supplementary material, further inquiries can be directed to the corresponding author.

## Footnotes

1
https://huttenhower.sph.harvard.edu/kneaddata/

2
http://huttenhower.sph.harvard.edu/kneaddata
