# Supplementary material for: Metagenomic insights into postbiotic-mediated modulation of strawberry surface microbiome and metabolic activity
Source: Front Microbiol. 2026 May 28;17:1841388. doi: 10.3389/fmicb.2026.1841388 (PMC13253626; doi:10.3389/fmicb.2026.1841388)
Supplement: Supplementary file 1 [file Table_1.docx]

**Supplementary materials**

**Figure S1.** Shotgun sequencing Workflow.


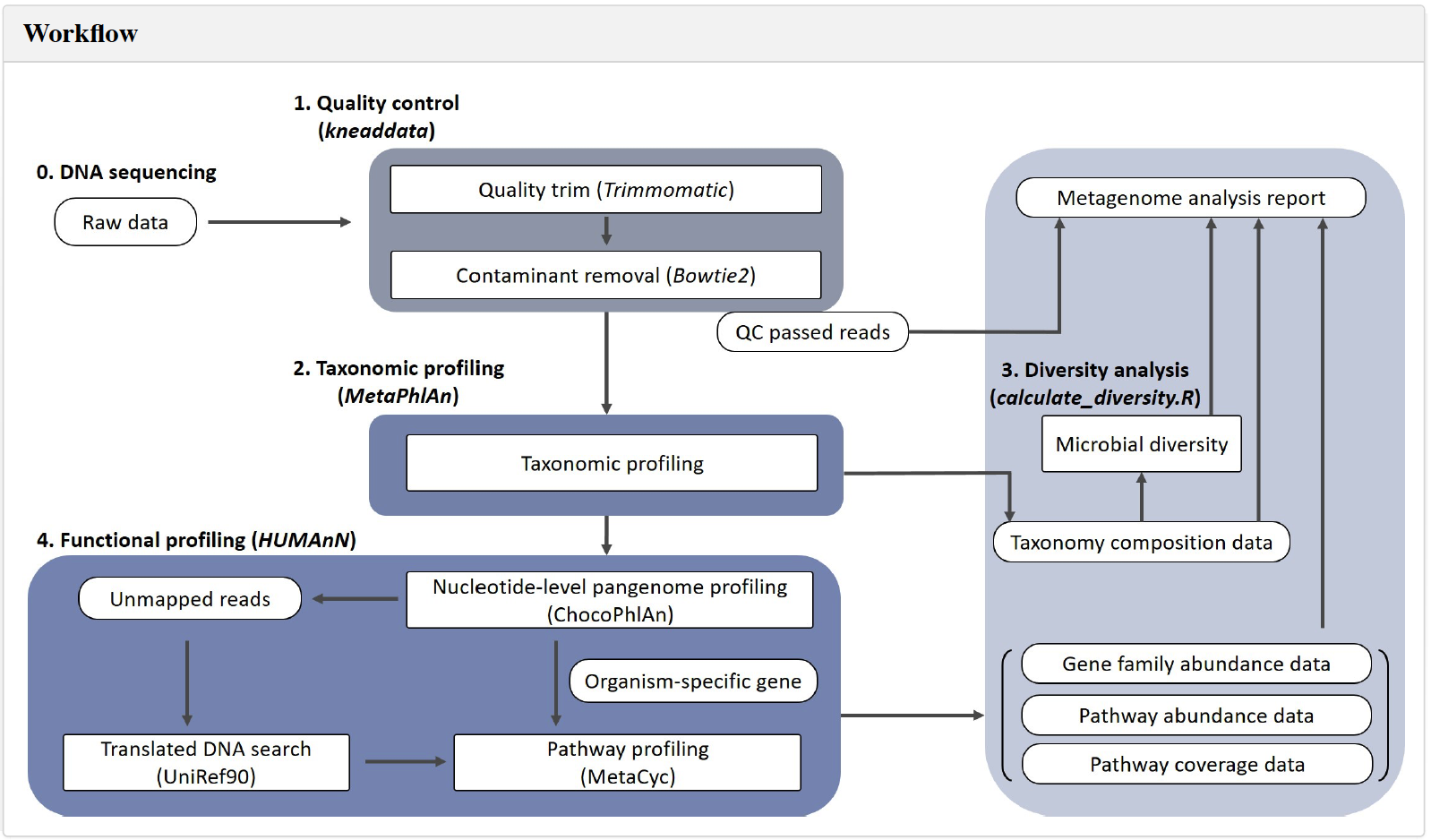


Table S1. Summary of shotgun metagenomic sequencing data processing across treatments.
For each sample (CD, FF1, FF2, FF3), the total number of raw reads, reads retained after adapter trimming, reads remaining after host ( *Fragaria × ananassa* ) removal, and the percentage of quality-controlled reads (Phred ≥ 20) are shown. High retention rates (>91%) across all samples indicate consistent sequencing quality and effective preprocessing. CD: commercial disinfectant; FF1: (1 x MIC, PPGt21O + EPSCys2-2: 1:1, v/v), FF2: (PPGt21O, 1 x MIC), FF3: (EPSCys2-2, 1 x MIC).

| **SAMPLE NAME** | **TOTAL NUMBER OF READS** | **TOTAL NUMBER OF TRIMMED READS** | **TOTAL NUMBER OF HOST REMOVAL READS** | **QC PASSED READS (%)** |
| --- | --- | --- | --- | --- |
| CD | 40,926,822 | 37,853,381 | 37,610,048 | 91.9 |
| FF1 | 40,982,900 | 37,856,018 | 37,693,448 | 91.97 |
| FF2 | 39,040,721 | 36,332,082 | 36,181,378 | 92.68 |
| FF3 | 38,948,994 | 36,074,686 | 35,936,587 | 92.27 |
